# Diagnosing psychiatric conditions in patients with cardiac symptoms: the role of cl psychiatry

**DOI:** 10.1192/j.eurpsy.2026.10359

**Published:** 2026-07-31

**Authors:** F. Novais, C. Siopa, D. Telles-Correia

**Affiliations:** Medicine, University of Lisbon, Lisbon, Portugal

## Abstract

**Background:**

Patients presenting with cardiac symptoms such as chest pain, palpitations, dyspnea, or syncope frequently exhibit underlying psychiatric conditions that may mimic or exacerbate cardiovascular disease. Anxiety disorders, depressive disorders, and somatic symptom disorders are highly prevalent in cardiology and emergency settings, yet remain underdiagnosed, contributing to repeated investigations, increased healthcare utilization, and poorer clinical outcomes. Consultation-liaison (C-L) psychiatry plays a pivotal role in the differential diagnosis and management of these complex presentations.

**Objectives:**

This presentation aims to review recent evidence on the prevalence, diagnostic challenges, and clinical impact of psychiatric disorders in patients with cardiac symptoms, and to highlight the contribution of C-L psychiatry to improving diagnostic accuracy and patient care.

**Methods:**

A narrative review of recent literature (last 3–5 years) indexed in PubMed was conducted, focusing on systematic reviews, meta-analyses, and large cohort studies examining psychiatric comorbidity in cardiac populations and the effectiveness of psychiatric assessment in cardiology and emergency contexts.

**Results:**

Recent studies indicate that up to 30–50% of patients presenting with non-cardiac chest pain meet criteria for an anxiety or depressive disorder, with panic disorder being particularly prevalent. Symptom overlap between cardiac and psychiatric conditions frequently leads to diagnostic uncertainty and delayed psychiatric referral. Evidence supports the use of structured screening instruments (e.g., GAD-7, PHQ-9) and early psychiatric consultation to enhance diagnostic clarity. C-L psychiatry interventions have been associated with reduced unnecessary cardiac testing, improved symptom management, and better patient-reported outcomes.

**Conclusions:**

Integrating C-L psychiatry into cardiology and emergency services is essential for the timely identification of psychiatric disorders in patients with cardiac symptoms. Early, structured psychiatric assessment represents a best-practice approach to reduce misdiagnosis, optimize treatment strategies, and improve both mental and cardiovascular health outcomes. Future research should focus on standardized clinical pathways and longitudinal outcomes of integrated care models.

**Disclosure of Interest:**

None Declared

